# The Spot‐Dam Technique: A Protocol for Adhesive Cementation of BOPT Crowns

**DOI:** 10.1111/jerd.70174

**Published:** 2026-05-04

**Authors:** Maria Giolanta Liaropoulou, Andrea Tinti, Roberto Padrós Roldan, Markus B. Blatz, Rui I. Falacho

**Affiliations:** ^1^ Master of Esthetic and Oral Rehabilitation University Polytechnical of Catalunya Barcelona Spain; ^2^ Department of Preventive and Restorative Sciences University of Pennsylvania School of Dental Medicine Philadelphia Pennsylvania USA; ^3^ ADEMA University School, University of the Balearic Islands (UIB) Palma Spain

**Keywords:** adhesive cementation, airborne‐particle abrasion, biologically oriented preparation technique, self‐adhesive resin cement, vertical preparation, zirconia

## Abstract

**Objective:**

The Biologically Oriented Preparation Technique (BOPT) uses vertical tooth preparation without a defined finish line, guided by provisional restorations to shape the gingival emergence profile. Subgingival crown margins inherent to BOPT increase the risk of residual cement and soft‐tissue trauma during adhesive cementation. This article presents the Spot‐Dam Technique (SDT), a continuous or staged isolation protocol designed to enhance visibility, facilitate excess cement removal, and protect peri‐gingival tissues in BOPT restorations.

**Clinical Considerations:**

SDT employs localized rubber dam fragments stabilized with polytetrafluoroethylene (PTFE) tape during water–airborne‐particle abrasion (WAPA) of dentin, followed by sulcular isolation during crown seating. Zirconia crowns were conditioned with air‐particle abrasion, and self‐adhesive resin cement was applied. Excess cement was removed at the gel stage to minimize soft‐tissue trauma. The protocol integrates soft‐tissue protection, dentin pretreatment, restoration surface conditioning, and controlled cement removal to optimize adhesion and margin integrity in subgingival restorations.

**Conclusions:**

The SDT provides a reproducible workflow for adhesive cementation of BOPT crowns. Isolation with rubber dam and PTFE tape protects peri‐gingival tissues, facilitates complete cement removal, and preserves seating accuracy, supporting biological stability and prosthetic predictability in vertically prepared teeth.

**Clinical Significance:**

Subgingival crown margins inherent to the BOPT increase the risk of sulcular cement retention and soft‐tissue trauma during definitive cementation. The isolation strategy described in the current manuscript combines sulcular displacement and contamination control to improve visibility, facilitate cement removal, and protect peri‐gingival tissues during adhesive cementation of indirect restorations to vertically prepared teeth.

## 1 Introduction

The Biologically Oriented Preparation Technique (BOPT) is based on vertical tooth preparation without a defined finish line, followed by immediate provisionalization, with the provisional restoration guiding the development of the gingival emergence profile [1]. The technique has been extensively described with respect to preparation geometry, provisional restoration design, and soft‐tissue response, and prospective clinical evidence supports favorable periodontal and prosthetic outcomes when the protocol is executed appropriately [2, 3].

Despite the emphasis placed on preparation and tissue conditioning, the definitive cementation phase has received comparatively less attention, even though it can critically influence both biological and mechanical outcomes. Cementation is particularly demanding in biologically oriented preparations because crown margins are intentionally positioned subgingivally, increasing the likelihood of excess cement retention. Residual cement has been associated with gingival inflammation, attachment loss, and long‐term biological complications, underscoring the importance of a controlled and predictable cementation protocol [4, 5].

Definitive cementation of biologically oriented crowns may be performed using either conventional luting agents or resin‐based cements. Conventional cements, such as glass ionomer or resin‐modified glass ionomer cements, may offer simplified handling and facilitate excess cement removal in specific situations; nevertheless, their long‐term stability is not always predictable, and cement dissolution has been implicated as a contributing factor to marginal breakdown and caries development [6]. Resin‐based cements, including self‐adhesive cements, provide increased retention and lower solubility, which may be advantageous in retreatment scenarios where remaining tooth structure is limited, a common indication for vertical preparations [6, 7]. At the same time, resin cementation is inherently technique‐sensitive and depends on reliable moisture control, which is challenging in the presence of subgingival margins [7].

For definitive BOPT crowns, zirconia‐based ceramics are often selected because their mechanical strength allows reduced minimum thicknesses without compromising structural integrity, which is relevant in subgingival preparations with limited space. In addition, zirconia's opacity and color stability support masking of dark substrates such as discolored dentin or post cores, whereas silica‐based glass ceramics may require greater thickness at the margin and provide less masking capacity [8]. From a biological standpoint, zirconia has been associated with favorable soft‐tissue response and low plaque affinity, which is clinically relevant when restoration margins are positioned within the sulcus [9].

Current bonding concepts for zirconia emphasize mechanochemical surface pretreatment and phosphate monomer‐based strategies, with systematic reviews indicating that self‐adhesive resin cements containing 10‐methacryloyloxydecyl dihydrogen phosphate (10‐MDP) may provide effective bonding to zirconia [10, 11]. In this context, practical adhesive workflows, such as the APC and AC protocols [12, 13], have been proposed to standardize zirconia pretreatment and reduce technique sensitivity. However, there is no universal consensus regarding optimal preparation of the tooth substrate, which is most often dentin in crown restorations.

Historically, slight bur roughening of the abutment immediately before cementation has been suggested to increase surface roughness and retention. Nevertheless, uncontrolled or irregular bur roughening may compromise crown seating through uneven cement thickness and internal stress concentration at the tooth‐cement‐restoration interface [14, 15]. In contrast, water–airborne‐particle abrasion (WAPA) has been reported to increase surface cleanliness, surface energy, wettability and micromechanical retention, and to improve bond strength compared with cleaning using pumice alone [16, 17]. These observations support the inclusion of a controlled dentin pretreatment step with WAPA, particularly in clinical situations where maximum bonding effectiveness is desired [17].

In BOPT restorations, crown margins are intentionally positioned within the gingival sulcus, making excess cement removal inherently more demanding and increasing the risk of residual subgingival cement. Adhesive cementation under these conditions presents distinct clinical challenges, including the need for effective abutment surface conditioning, isolation, and moisture control in the presence of subgingival margins. These constraints increase the likelihood of contamination during cementation and may compromise the quality and longevity of the adhesive interface if not managed through a standardized, reproducible workflow.

Retraction cords have been placed within the sulcus to displace soft tissue, control gingival crevicular fluid, and provide access to subgingival finish lines during crown seating and cementation. However, a single retraction cord is generally insufficient for vertically prepared BOPT abutments because it often fails to provide the horizontal tissue displacement required to achieve circumferential sulcular isolation and predictable cement control. A technique involving polytetrafluoroethylene (PTFE/Teflon) tape for relative isolation of a prepared tooth during adhesive cementation has been described to limit exposure of the bonding area to saliva and gingival crevicular fluid and to facilitate cement control in situations where absolute isolation with a rubber dam may interfere with crown seating in deeply subgingival margins [18, 19].

In this context, the Spot‐Dam Technique (SDT) is proposed as a localized rubber dam isolation approach designed to isolate a single or, in specific cases, multiple abutment teeth during critical adhesive steps while preserving access to subgingival margins and avoiding interference with definitive crown seating. SDT combines a rubber dam fragment, stabilized by PTFE tape, during WAPA, followed by either maintenance of the rubber dam fragment or replacement with circumferential PTFE sulcular isolation during crown seating and excess cement control.

To date, the literature lacks detailed, clinically oriented protocols specifically addressing adhesive cementation in BOPT restorations. The absence of standardized recommendations may contribute to variability in clinical execution and reduce predictability. Therefore, a clearly defined step‐by‐step technique that integrates soft‐tissue management, abutment surface preparation, and contamination control strategies tailored to the biological and prosthetic principles of BOPT is needed. Accordingly, the purpose of this article is to present SDT as a detailed clinical protocol for adhesive cementation of BOPT crowns, integrating localized rubber dam protection for abutment pretreatment in combination with possible PTFE‐based sulcular isolation for cementation and excess cement removal, with the goal of supporting peri‐gingival tissue stability, biological integration, and prosthetic predictability.

## 2 Clinical Technique

### 2.1 Indications

The SDT protocol is indicated for adhesive cementation of definitive crowns on vertically prepared abutments with margins extending within the gingival sulcus, where predictable contamination control and complete cement removal are critical.

### 2.2 Rationale

The protocol combines:

- Soft‐tissue protection, allowing dentin WAPA without sulcular disruption or bleeding;
- Effective isolation from crevicular fluid and any sulcular exudates;
- Controlled soft‐tissue displacement to facilitate access to subgingival areas during crown seating and cementation;
- Circumferential PTFE sulcular isolation during seating, followed by cement removal at the viscous stage to ensure complete elimination of excess cement.

### 2.3 Prerequisites and Limitations

Stable peri‐gingival tissues, adequately conditioned by the provisional restoration, must be established before cementation. This technique description aims to standardize key cementation steps rather than to provide long‐term outcome data.

### 2.4 Materials and Products Used

Materials used in the clinical case are summarized in Table 1.

**TABLE 1:** Materials used in the clinical case.

Material	Classification	Manufacturer
GC Unifast LC A2	Acrylic resin	GC America, Chicago, IL, USA
OptiBond FL	Adhesive system (etch‐and‐rinse)	Kerr, Orange, CA, USA
27‐μm KaVo RONDOflex preparation powder aluminum oxide	Air‐abrasion powder	KaVo Dental, Biberach an der Riß, Germany
50‐μm KaVo RONDOflex preparation powder aluminum oxide	Air‐abrasion powder	KaVo Dental, Biberach an der Riß, Germany
LM‐Arte composite instrument kit	Composite instrument kit	LM‐Instruments Oy, Parainen, Finland
Empress direct A2D	Conventional composite resin (build‐up)	Ivoclar Vivadent, Schaan, Liechtenstein
KSK rubber dam punch	Dam Punch	Dentech Corporation, Itabashi‐ku, Tokyo, Japan
Preparation drill kit; Sweden & Martina	Diamond burs	Sweden & Martina, Due Carrare, Italy
IPS classic V feldspathic ceramic	Feldspathic ceramic	Ivoclar Vivadent, Schaan, Liechtenstein
Tetric EvoFlow A2 Syringe	Flowable composite	Ivoclar Vivadent, Schaan, Liechtenstein
Gel‐Cord 25% aluminum sulfate gel	Hemostatic gel	Pascal International, Bellevue, WA, USA
Medit i900	Intraoral scanner	Medit, Seoul, South Korea
TEFLODENT PTFE Tape 0.076 mm	PTFE tape	Innova Dentale S.r.l.s., Rome, Italy
Ultrapak 000 & 0	Retraction cord	Ultradent Products, South Jordan, UT, USA
Nic Tone Dental Dam	Rubber dam (medium gauge)	MDC Dental, Zapopan, Jalisco, Mexico
KaVo RONDOflex Plus 360	Sandblasting device	KaVo Dental, Biberach an der Riß, Germany
PANAVIA SA Cement Universal	Self‐adhesive resin cement	Kuraray Noritake Dental, Tokyo, Japan
Teflon Packer 1.2 mm	Teflon Packer	LM‐Instruments Oy, Parainen (Pargas), Finland
IPS e.max ZirCAD Prime C17	Zirconia Block	Ivoclar Vivadent, Schaan, Liechtenstein

### 2.5 Clinical Presentation and Diagnostic Assessment

**FIGURE 1:**
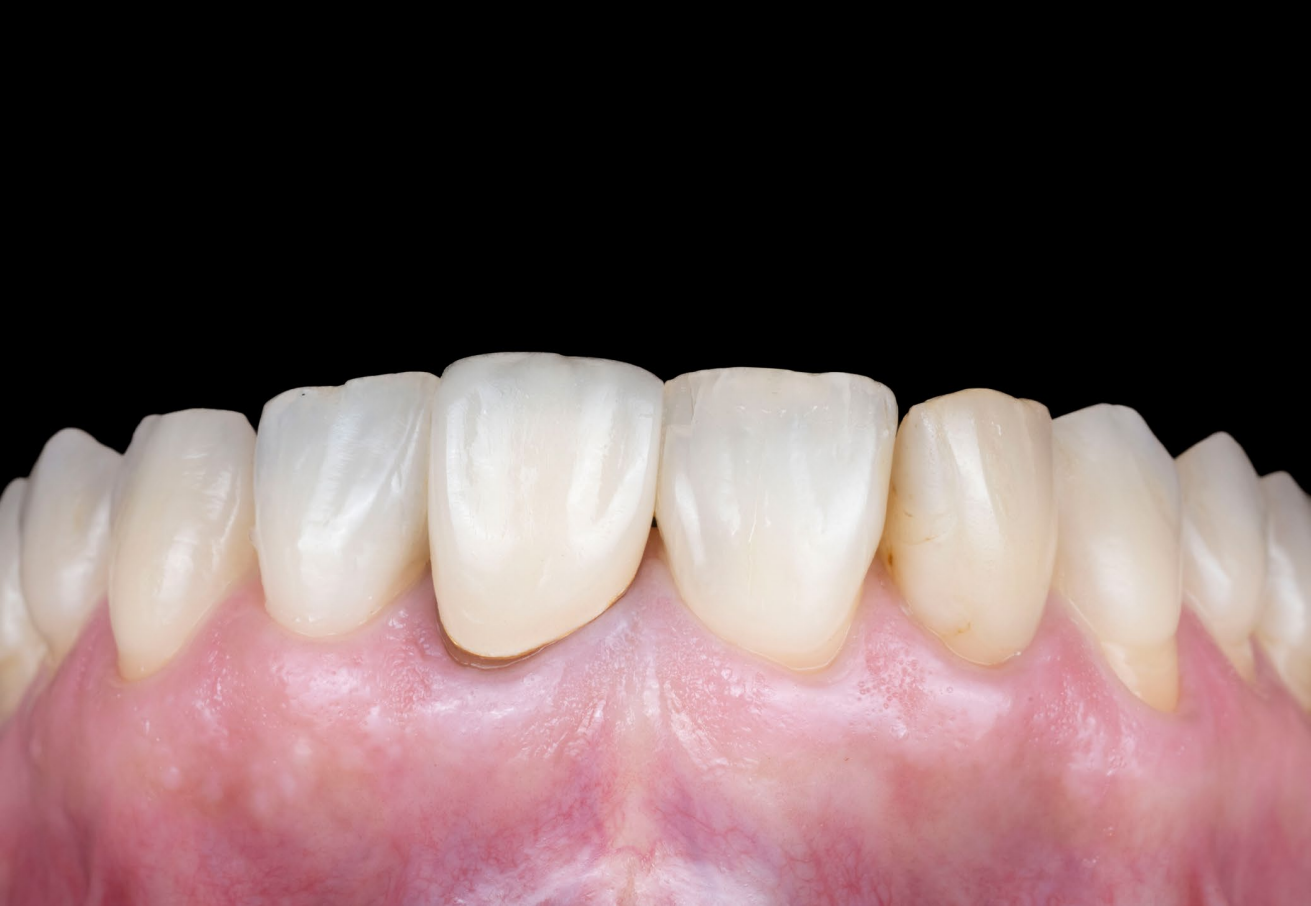
Initial situation.

**FIGURE 2:**
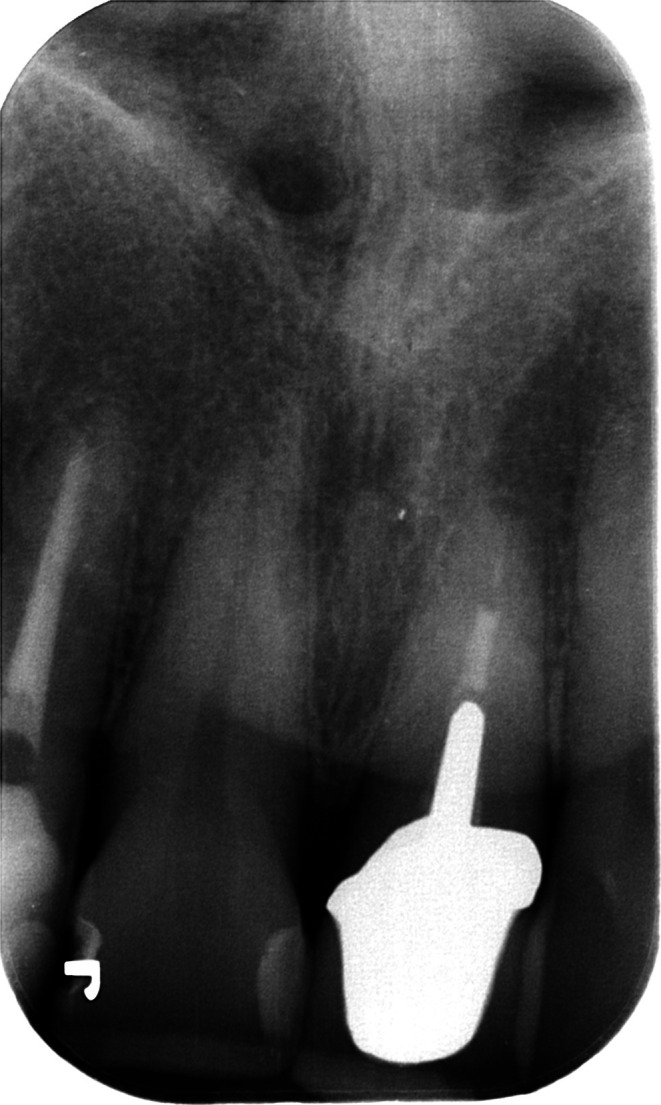
Initial radiographic examination.

A patient sought improvement of smile esthetics, as the existing crown on the upper left central incisor differed in appearance from the contralateral incisor. At the initial appointment, comprehensive information was collected through a clinical anamnesis, intraoral examination, radiographic assessment, and intraoral and extraoral photographs. Clinical findings included localized gingival discoloration and recession at the maxillary left central incisor and a crown with chroma, opacity, and proportions that did not match the contralateral tooth (Figures 1 and 2). Radiographic examination revealed a previous endodontic treatment and a metallic post, without clinical signs or symptoms of periapical inflammation.

The existing endodontic treatment and metallic post were maintained to minimize the risk of fracture associated with post removal. Although the ideal treatment option would have been endodontic retreatment with internal bleaching, followed by placement of a new post and core, an approach considered the most effective for achieving a tissue color closer to that of the contralateral tooth, this option was not pursued due to the associated risks. For overall esthetic harmonization, existing composite restorations on the maxillary right lateral and central incisors were replaced, an internal bleaching was performed after endodontically treating the maxillary right lateral incisor to increase its value.

Considering the presence of gingival recession, and underlying root discoloration, a BOPT was selected. The primary objective was to increase soft tissue thickness to better mask the discoloration of the root. Subsequently, the approach aimed to establish more stable soft tissues over time, enhancing long‐term tissue stability. Additionally, BOPT enabled optimization of the emergence profile and harmonization of gingival contours through guided soft tissue healing with provisionalization.

### 2.6 Step‐by‐Step Protocol

#### 2.6.1 Step 1. Crown Removal and Composite Build‐Up

**FIGURE 3:**
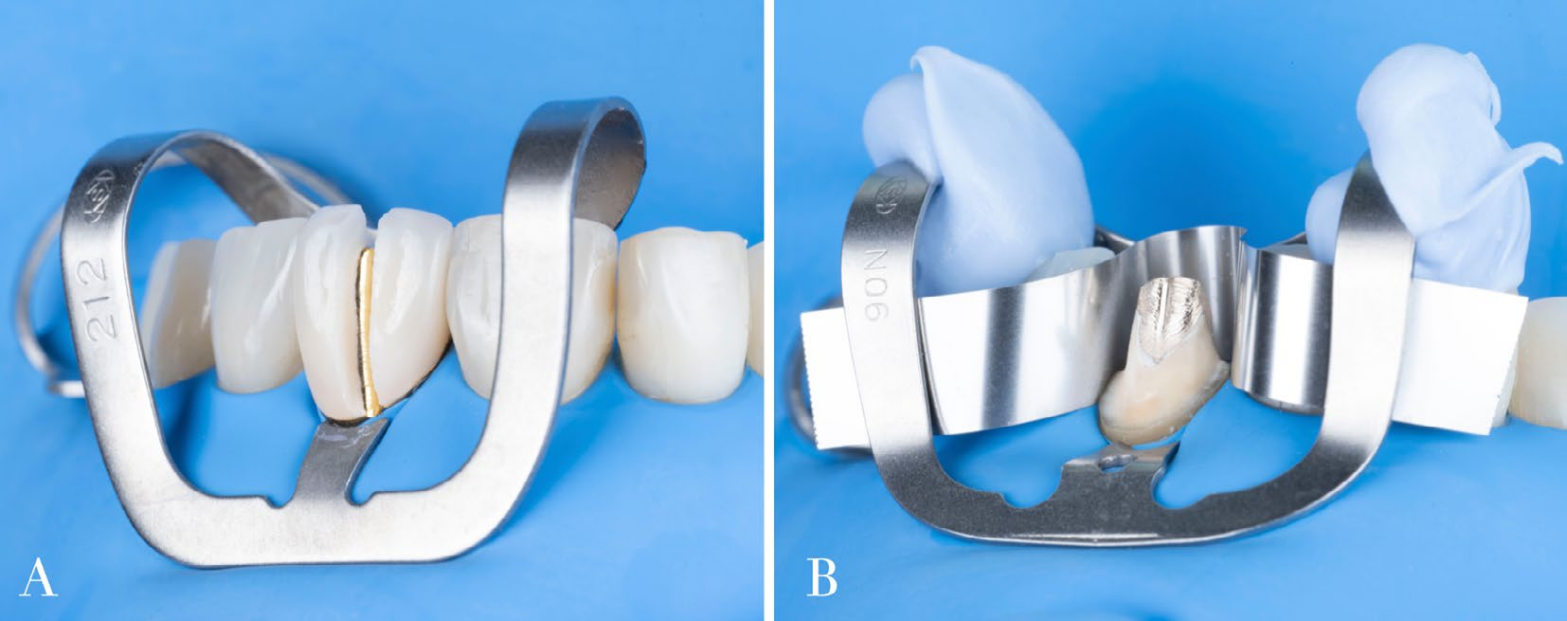
(A) Crown removal; (B) abutment pretreatment.

The existing metal‐ceramic crown was removed. A composite build‐up (Empress Direct A2D; Ivoclar Vivadent, Schaan, Liechtenstein) was performed under rubber dam isolation (Nic Tone Dental Dam; MDC Dental, Zapopan, Jalisco, Mexico) to reinforce the abutment and achieve a less invasive vertical preparation, as well as mimic the dentin substrate with a biomechanically equivalent restorative material (Figure 3). Following crown removal, two main discrepancies relative to the contralateral tooth were observed: (i) the mesiodistal space was noticeably narrower and (ii) the abutment was positioned more buccally. To improve symmetry, enameloplasty of the adjacent lateral incisor was performed, and additional buccal reduction was required to create appropriate space for the intended buccal contour.

#### 2.6.2 Step 2. Vertical Preparation, Transition to BOPT, and Provisional Restoration

**FIGURE 4:**
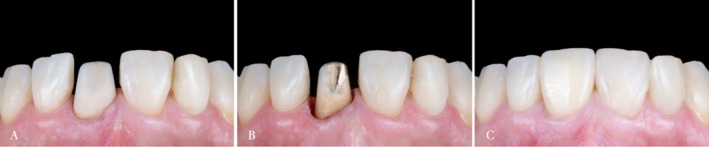
(A) Vertical preparation; (B) BOPT preparation; (C) provisional follow‐up.

A vertical preparation was performed initially without subgingival extension (Figure 4A). After confirming adequate prosthetic space, the provisional crown was rebased. Subsequently, a BOPT preparation was carried out by angulating the bur subgingivally, guided by the root inclination, to eliminate the previous finishing line and create space for soft tissue. Undercuts were removed by preparing along the long axis of the tooth, and the preparation was polished. A provisional restoration was fabricated with acrylic resin (GC Unifast LC A2; GC America, Chicago, IL, USA) and relined intraorally (Figure 4B,C).

**FIGURE 5:**
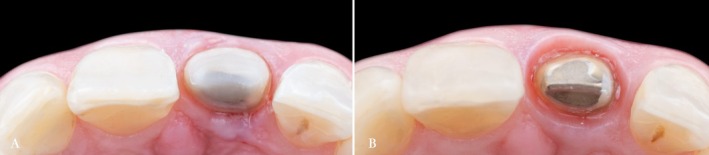
(A) Soft tissues before BOPT; (B) soft tissues after BOPT at 6 weeks of follow‐up.

**FIGURE 6:**
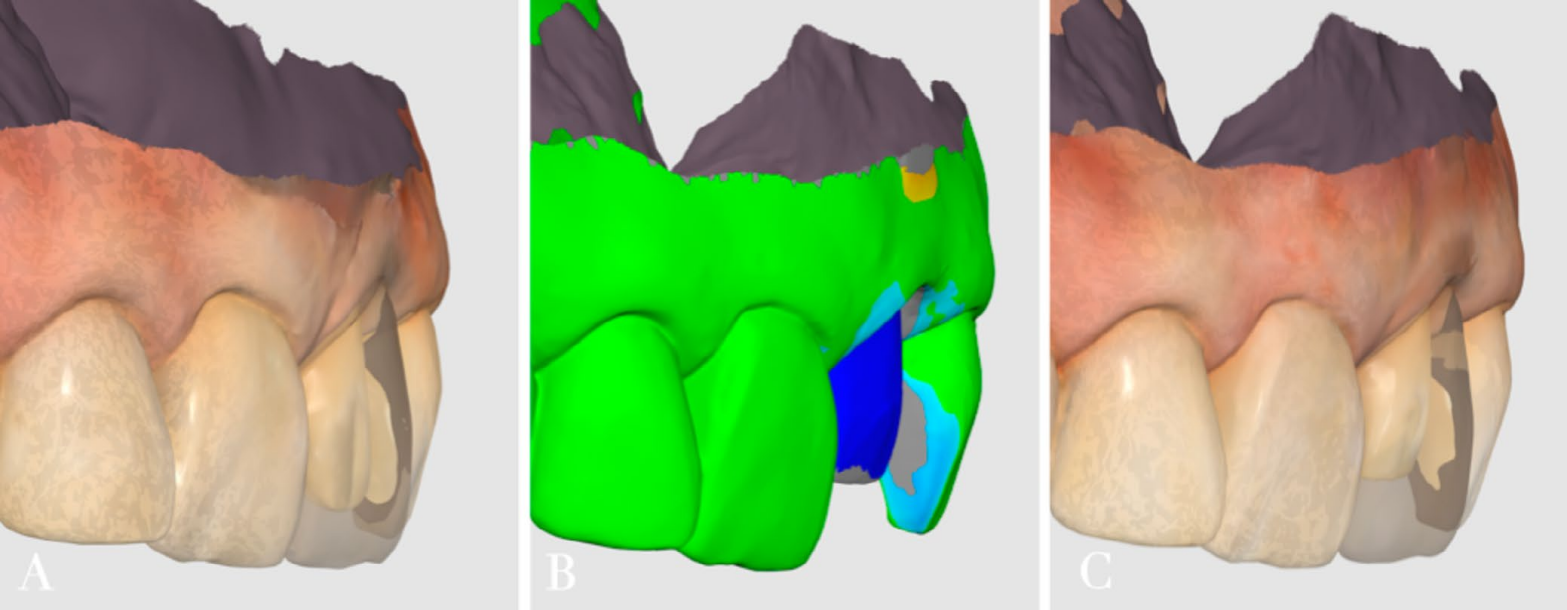
(A) The abutment before any modification; (B) digital analysis of the prosthetic space after incisal and buccal reduction; (C) the abutment after modification.

To establish a new prosthetic emergence profile and stabilize the blood clot consistent with the technique concept, flowable composite resin (Tetric EvoFlow A2 Syringe; Ivoclar Vivadent, Schaan, Liechtenstein) was added extraorally to the provisional restoration after relining. The soft tissues were allowed to heal for a minimum of 4 weeks before proceeding to the final impression (Figure 5). During the final impression appointment, the abutment was further modified to create additional buccal space following digital analysis of the available prosthetic spaces (Medit design application from Medit i900; Medit Corp., Seoul, South Korea), which also required occlusal modification due to insufficient thickness (Figure 6).

#### 2.6.3 Step 3. Prototype‐Guided Planning and Definitive Restoration Fabrication

**FIGURE 7:**
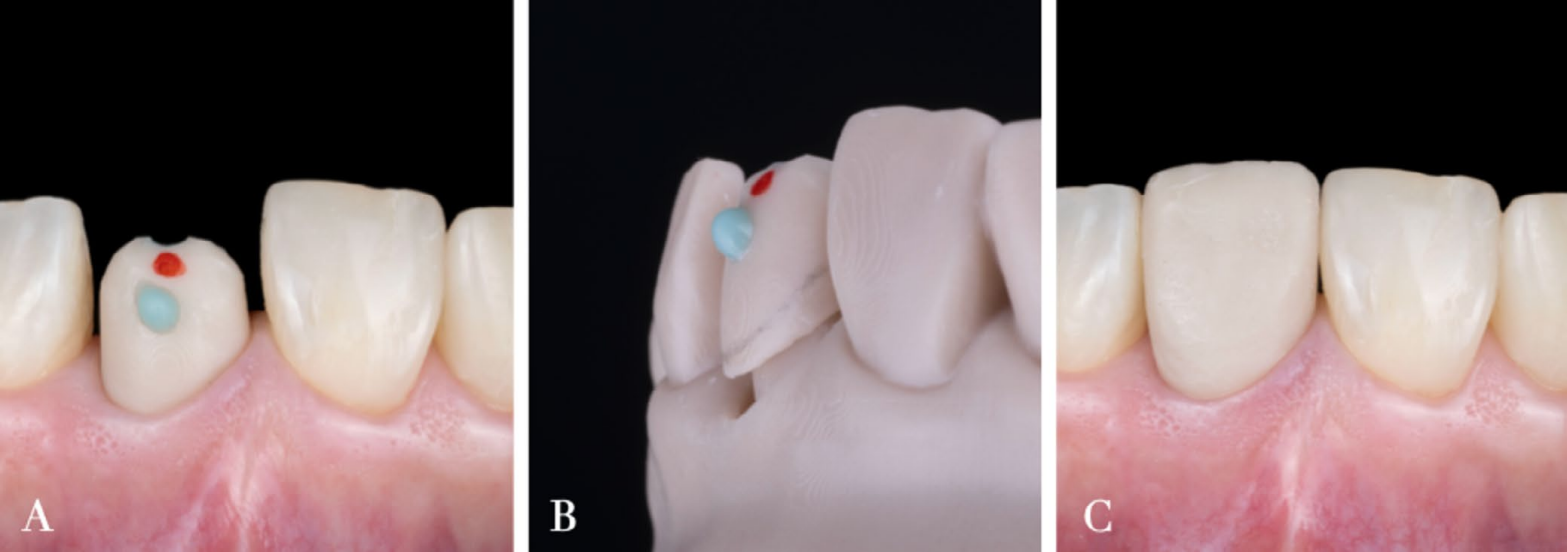
Prototype for (A) margin intraorally; (B) margin on model; (C) shape.

After transfer of clinical and digital records to the laboratory, three‐dimensional (3D) printed prototypes were designed and manufactured following the protocol described in the literature [20] to improve predictability of the definitive restoration (Figure 7). The final ceramic restoration was milled from a zirconia block and characterized by feldspathic ceramic layering on the facial surface only, reflecting the esthetic demands of a single maxillary central incisor restoration.

#### 2.6.4 Step 4. Try‐In

**FIGURE 8:**
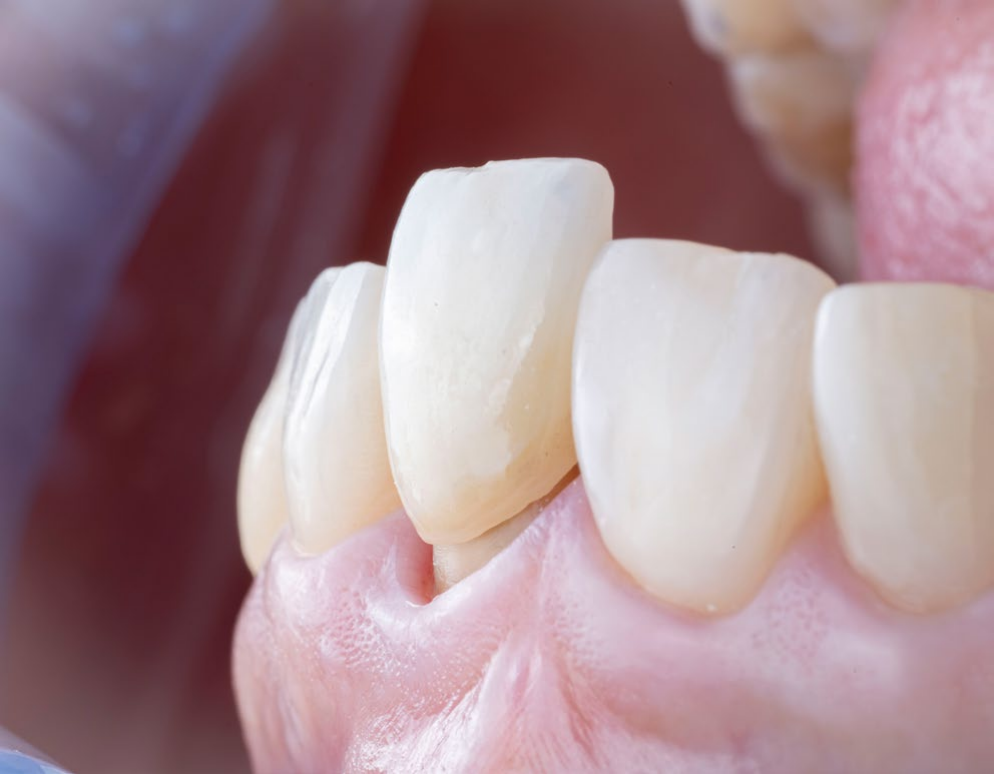
Try‐in of the final restoration.

At the cementation appointment, the provisional restoration was removed, the abutment was cleaned of temporary cement remnants and plaque using an ultrasonic instrument and a brush with pumice paste, avoiding contact with the gingiva to prevent bleeding. Try‐in of the definitive restoration was performed to verify fit, proximal contact, occlusion, and subgingival extension (Figure 8).

#### 2.6.5 Step 5. Field Stabilization Before Substrate Pretreatment

**FIGURE 9:**
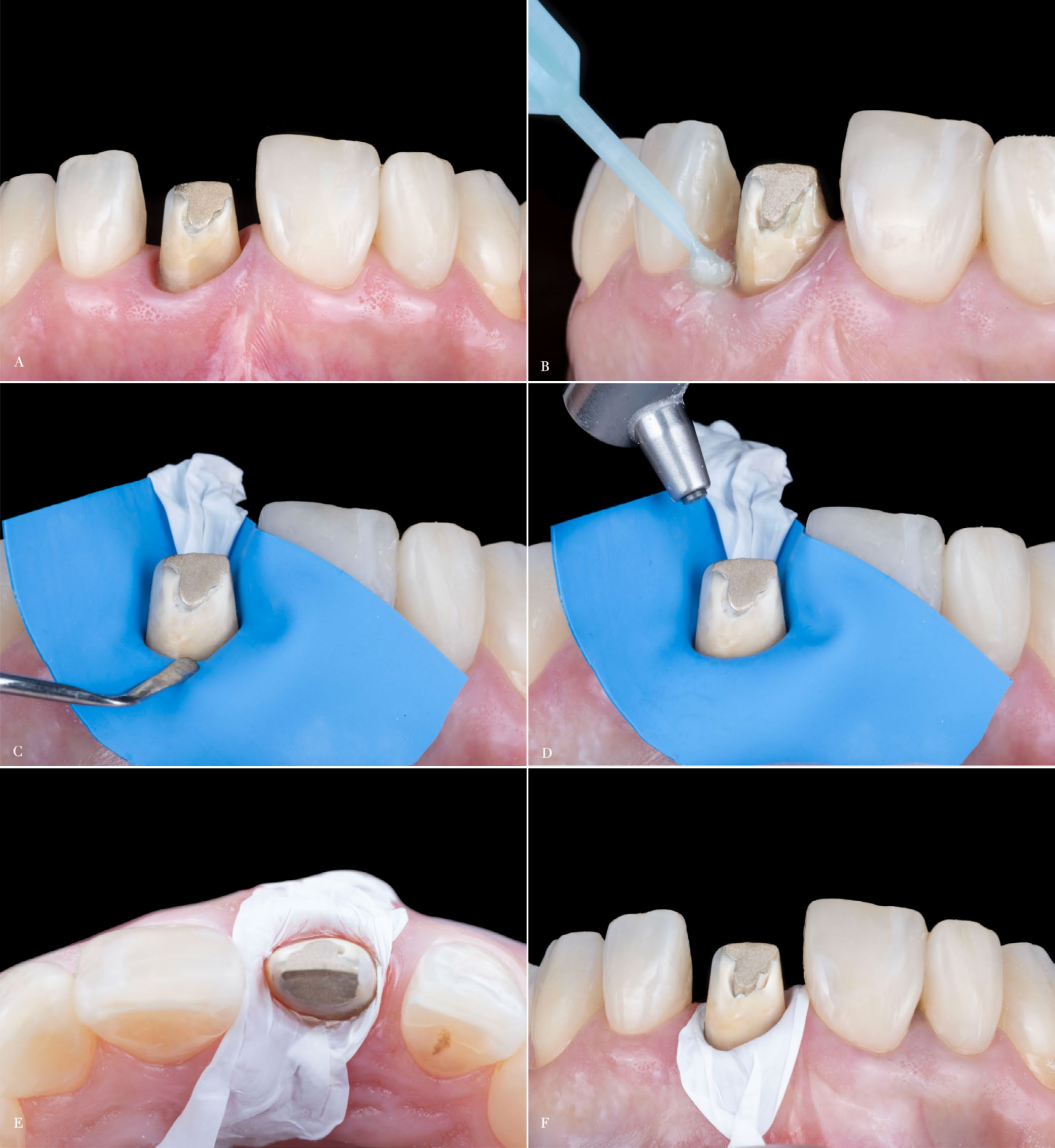
Pretreatment of tooth substrate: (A) Initial situation; (B) intrasulcular hemostatic gel; (C) placement of rubber dam with spatula and Teflon tape for fixation; (D) water‐airborne‐particle abrasion; (E) Teflon tape positioned for sulcular isolation; (F) abutment ready for cementation.

A hemostatic gel (Gel‐Cord 25% Aluminum Sulfate Gel; Pascal International, Bellevue, WA, USA) was applied for a minimum of 3 min to control bleeding and/or sulcular fluid and obtain a stable, dry field during cementation. The gel was then thoroughly rinsed with water, and the field air‐dried (Figure 9A,B).

#### 2.6.6 Step 6. SDT

In this protocol, the most challenging and technique‐sensitive step of the cementation procedure is pretreatment of the tooth substrate with WAPA while the surrounding soft tissues must remain protected, without bleeding and the operative field must remain stable.

A small segment of rubber dam (Nic Tone Dental Dam Medium; MDC Dental, Zapopan, Jalisco, Mexico) was trimmed with scissors, and a perforation was created at its center using the smallest hole of a rubber dam perforator. The segment was then adapted around the abutment using a spatula (Teflon Packer 1.2 mm; LM‐Instruments Oy, Parainen (Pargas), Finland). This segment alone does not provide adequate tooth exposure at the sulcular area. To overcome this limitation, a small piece of polytetrafluoroethylene (TEFLODENT PTFE Tape 0.076 mm; Innova Dentale S.r.l.s., Rome, Italy) tape was trimmed and gently inserted into the sulcus using a teflon packer instrument, in a manner comparable to cord packing (Figure 9C). When additional external stabilization of the rubber dam is required to prevent displacement under the force of the WAPA stream, at which stage the rubber dam may flap, cover the tooth, or become dislodged, a small drop of cyanoacrylate (PeriAcryl; GluStitch Inc., Delta, British Columbia, Canada) can be applied to its extremity, and the rubber dam gently pressed against the palatal and vestibular soft tissues, avoiding contact of cyanoacrylate with dental surfaces. After a few seconds, the cyanoacrylate provides stable fixation of the rubber dam; this stabilization is easily reversible and can be removed without difficulty. This maneuver stabilizes the rubber dam while effectively protecting the peri‐gingival tissues and adjacent teeth during the following procedure.

#### 2.6.7 Step 7. WAPA

WAPA was performed after the SDT for protection of the soft tissues, using an air‐abrasion device (KaVo RONDOflex Plus 360; KaVo Dental, Biberach an der Riß, Germany) at 2–4 bar with 27 μm aluminum oxide particles. The nozzle was positioned approximately 5–10 mm from the surface and maintained in continuous motion, with the goal of surface cleaning and micro‐roughening rather than material removal (Figure 9D). After air abrasion, the dentin was thoroughly rinsed to eliminate residual particles and air‐dried, leaving the surface dry and conditioned for the subsequent adhesive cementation protocol.

#### 2.6.8 Step 8. Isolation Strategy Assessment

The clinician may choose to leave the rubber dam fragment and PTFE tape in place when it is certain that they do not interfere with restoration seating, or alternatively, remove them before proceeding, as the main objective has already been achieved. In the reported case, both the PTFE tape and the rubber dam fragment were carefully removed. The soft tissues remained dry, without evidence of fluid or bleeding.

The field was gently air‐dried, and a new PTFE segment was trimmed and perforated using a rubber dam perforator, then adapted around the abutment using a teflon packer instrument to achieve circumferential sulcular isolation without leakage while maintaining unimpeded restoration seating, as well as vertical and horizontal protection of the soft tissues (Figure 9E,F).

In conclusion, after WAPA, two isolation strategies were considered depending on clinical conditions.

1. **Retention strategy:** the rubber dam fragment and PTFE tape may be left in place if they do not interfere with restoration seating.
2. **Reset strategy:** both the rubber dam fragment and PTFE tape may be removed and a new PTFE‐based isolation reestablished prior to cementation.

#### 2.6.9 Step 9. Cement Selection

A self‐adhesive resin cement (PANAVIA SA Cement Universal; Kuraray Noritake Dental, Tokyo, Japan) was selected. According to the manufacturer's recommendations and the available scientific evidence, the use of a separate etchant, primer, or bonding agent on the tooth structure is not required, as bonding is based on phosphate‐monomer chemistry.

#### 2.6.10 Step 10. Restoration Surface Treatment

After the try‐in, the restoration was cleaned in an alcohol ultrasonic bath, dried, and the zirconia intaglio surface was air‐particle abraded (AC–Step A) using 50 μm alumina particles at up to 2 bar. The restoration was then thoroughly air‐dried.

#### 2.6.11 Step 11. Cementation

**FIGURE 10:**
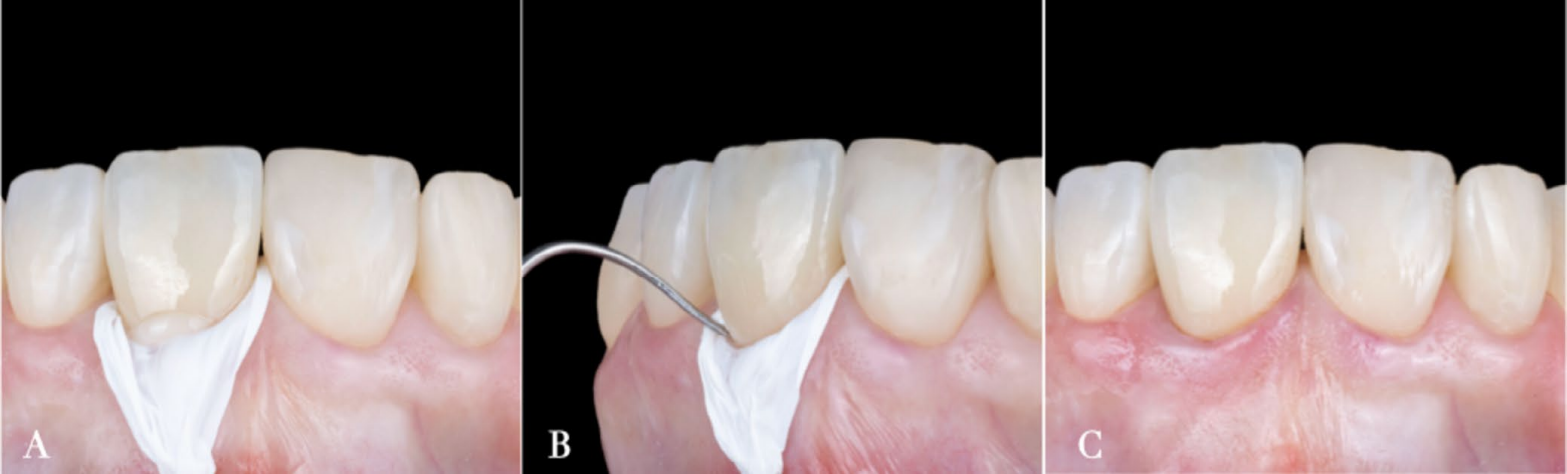
Cementation: (A) Excess of cement; (B) gel phase cement removal; (C) post‐cementation inspection.

Once the ceramic intaglio surface was conditioned, an appropriate amount of self‐adhesive cement was placed inside the crown, on the axial walls (AC–Step C). The crown was then seated intraorally, while the PTFE tape remained in place to protect the soft tissues. Owing to the vertical preparation design, which lack horizontal gaps, a tack‐curing technique was employed. A controlled, brief light‐activation (3 s each on the vestibular and palatal surfaces) was applied to bring the resin cement to a gel phase, thereby enabling clean and controlled removal of excess cement with a simple probe, without risk to the surrounding tissues (Figure 10A,B).

Final light curing was performed and PTFE tape was then gently lifted with an explorer and cut at the center to permit removal in two separate pieces. Any residual cement was carefully removed with a scaler. The area was rinsed and dried, and all margins were inspected to verify complete removal of excess cement.

The use of rubber dam or PTFE tape combined with removal of excess cement during its gel‐stage results in a clean working field, with the surrounding soft tissues remaining nearly intact (Figure 10C).

#### 2.6.12 Step 12. Follow‐Up

**FIGURE 11:**
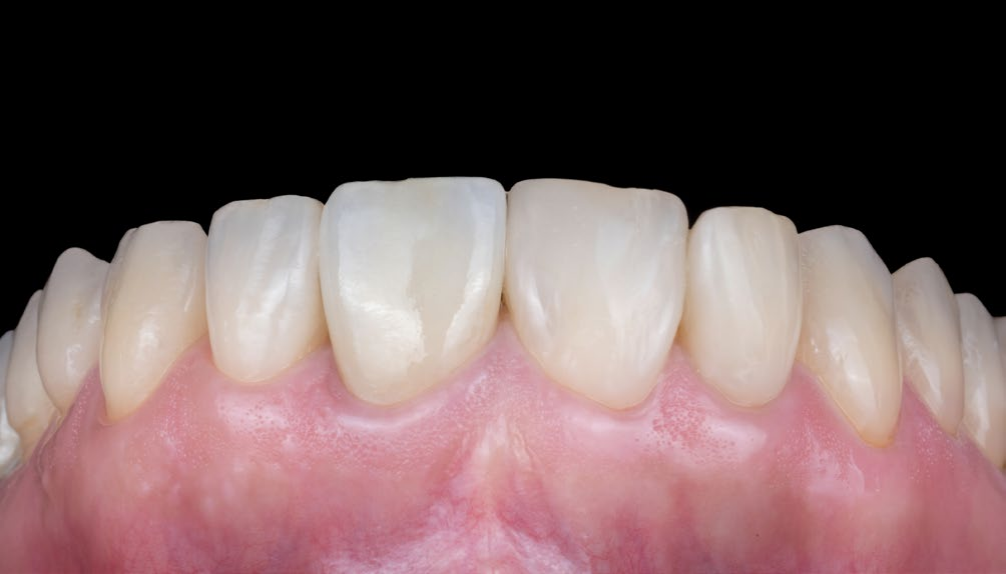
Final situation at one‐month follow‐up.

At one‐month follow‐up, the periodontal tissues demonstrated a favorable response without signs of inflammation. Esthetic integration of the restoration was observed, including masking of the discolored root substrate and patient‐reported satisfaction (Figure 11).

## 3 Discussion

This article presents a standardized clinical workflow for adhesive cementation of BOPT crowns and introduces the SDT, a localized rubber dam isolation approach designed to isolate a single abutment while preserving access to sulcular margins during adhesive procedures. Although the BOPT has been extensively described with respect to preparation design, provisionalization, and soft‐tissue conditioning, the definitive cementation phase has received comparatively less procedural standardization, despite its relevance to both biological and mechanical outcomes in restorations with intentionally subgingival finishing.

Adhesive cementation is frequently selected in BOPT cases, particularly in retreatment scenarios with compromised remaining tooth structure. Compared with conventional luting agents, resin‐based cements have been reported to provide increased micromechanical retention and lower solubility over time [5, 6, 7]. Conventional cements are more susceptible to hydrolytic degradation, particularly in restorations with subgingival extension, where the cement interface is exposed to crevicular fluid and biofilm, cement stability and marginal sealing are clinically relevant, and the potential biological impact of residual cement further increases the importance of cementation control. Adequate sulcular isolation also reduces gingival bleeding and moisture contamination, further enhancing the predictability of bonding procedures. At the same time, resin cementation remains technique‐sensitive and depends on predictable contamination control; therefore, the primary clinical challenge is not the selection of an adhesive cement alone but the ability to execute a reproducible protocol under subgingival conditions.

Isolation and cement control are central limitations in the currently performed adhesive cementation techniques of BOPT crowns. Conventional retraction cord techniques alone often fail to provide sufficient horizontal tissue displacement or circumferential isolation in vertical preparations. The adjunctive use of PTFE tape is a practical strategy to protect the bonding area from saliva and sulcular fluid while facilitating cement control in subgingival preparations. Absolute isolation with rubber dam, clamps, or floss ligatures represents the ideal condition for adhesive procedures; however, in BOPT restorations, this approach is frequently impractical or technically unfeasible. The absence of a defined finishing line and the anatomy of vertically prepared abutments often prevent stable clamp positioning, while the presence of isolation devices may interfere with complete crown positioning. SDT extends this concept by combining staged, localized rubber dam isolation with circumferential PTFE sulcular management. Specifically, a small rubber dam fragment, stabilized by PTFE tape, is used during the phase that requires the highest level of soft‐tissue protection, WAPA, thereby shielding the sulcus and adjacent tissues from abrasive particles.

However, selective isolation in BOPT cases is inherently technique‐sensitive, particularly due to the presence of newly formed junctional epithelium and connective tissue attachment following the preparation. In this context, meticulous tissue management, atraumatic handling, and adequate operator experience are essential to prevent disruption of the healing soft tissues and to ensure predictable clinical outcomes. When PTFE tape placement is not feasible in the most critical subgingival areas, close to the gingival margin, the rubber dam can be stabilized using two spatulas positioned on the vestibular and palatal/lingual aspects of the abutment. This approach allows adequate retraction and exposure of the substrate while maintaining protection of the surrounding soft tissues.

During seating and cementation, the rubber dam fragment may be removed if causing interference with complete crown seating. If rubber dam is removed, PTFE tape may be placed, as described in the current paper, to provide circumferential sulcular isolation, soft‐tissue displacement, and a controlled margin‐access window for cement cleanup. The intent of this staged approach is to maximize tissue protection and contamination control when most critical, while preserving seating accuracy and the biological concept of BOPT.

When compared with previously described PTFE‐based relative isolation workflows for BOPT restorations [18], important procedural distinctions merit consideration. In one described approach [18], the dentin substrate is cleaned using prophylaxis paste and water without additional mechanical pretreatment, which may limit the effectiveness of adhesion, particularly in areas requiring optimal bond strength. In contrast, the present protocol includes controlled WAPA, a step reported to increase surface cleanliness, micromechanical retention, and improve adhesive performance compared with pumice cleaning alone [16]. From a practical standpoint, SDT also targets the most vulnerable zone for contamination and iatrogenic trauma: the immediate sulcular margin during substrate pretreatment. By using a localized rubber dam fragment stabilized with PTFE during air abrasion, the protocol aims to protect the sulcular tissues while maintaining field stability.

A selective adhesive luting approach has also been described for situations in which placement of a conventional rubber dam before cementation is not feasible [21]. However, it differs from the current protocol as the present protocol incorporates WAPA as a standardized pretreatment step and uses circumferential sulcular isolation during both pretreatment and seating, whereas a lack of sulcular protection may increase the risk of contamination at the margin in clinical conditions that are not fully reproduced by in vitro models [17, 21]. Importantly, SDT is compatible with variations in tooth‐side adhesive strategies used by other authors, meaning that if an operator selects to incorporate an additional adhesive step on dentin, such an approach could be performed under the same isolation conditions described in the SDT technique, provided that field stability and seating accuracy are maintained.

This article does not aim to provide long‐term outcome data and should be interpreted as a technique description intended to improve reproducibility and biological respect during cementation of restorations with subgingival margins. The primary strength of SDT lies in the standardization of operator‐dependent steps that are commonly associated with variability—restorative and abutment surface preparation, isolation, and excess cement control—within a workflow designed to preserve crown seating accuracy. Future clinical studies with adequate sample sizes and follow‐up are required to evaluate biological stability and prosthetic durability of different adhesive cementation protocols in BOPT restorations.

Overall, the SDT provides a clinical practical approach for adhesive cementation of BOPT crowns, intended to minimize contamination, facilitate excess cement removal, and reduce the risk of soft‐tissue inflammation.

## 4 Conclusions

Within the limitations of this article, it was concluded that:

1. The SDT, a localized rubber dam isolation approach stabilized with PTFE tape, provided soft‐tissue protection during dentin WAPA before cementation of vertically prepared teeth with subgingival margins.
2. Circumferential PTFE sulcular isolation during seating provided margin access for controlled excess cement removal around subgingival crown margins.
3. A standardized workflow integrating soft‐tissue management, tooth substrate pretreatment, restoration internal surface conditioning, and gel‐stage excess cement removal established a reproducible protocol for adhesive cementation of BOPT crowns.

## Conflicts of Interest

The authors declare no conflicts of interest.

## Data Availability

The clinical data supporting the findings of this article are available from the corresponding author upon reasonable request. No publicly available dataset was generated or analyzed for this clinical technique article.
